# Ethics in occupational medicine

**DOI:** 10.4103/0019-5278.58911

**Published:** 2009-12

**Authors:** J. Kumar

**Affiliations:** Ocupational Health Consultant 24, Amprapali Arcade, 1^st^ Floor, Vasant Vihar, Thane, 400610 E-mail: kssg@vsnl.com

Ethics, as a principle, is concerned with how people behave when they are faced with a conflict between two or more principles to which they subscribe. In Occupational Medicine, the doctor is concerned with the management, the workforce and his profession as a doctor. In most of the conflicts there is no single correct solution and the doctor must make up his mind to arrive at the best possible solution.

Earlier, the main reasons for employment of doctors in the industry were primarily philanthropic, later, to avoid recruitment of the less healthy, but after the passage of The Workmen's Compensation Act of 1981, it was desired by the Management to protect its interest, either to avoid claims or to help the employer defend himself against the claims of sick or injured employees.

This editorial endeavors to look at some ethical issues in the practice of Occupational Medicine. The right perception of the occupational physician should be that of an independent professional adviser both to the management and the workers. An important aspect of ethics for an occupational physician is the confidentiality of medical information.

If consultation has been initiated by the employee, the information should be kept in strict confidence. On the other hand, if the management has requested that a medical examination be done to assess the employee's eligibility to retire on a disability pension, the result of the examination can be passed on to the management and the doctor should convey this to the employee before and after the examination.

## PRE-EMPLOYMENT AND STATUTORY MEDICAL EXAMINATION

The doctor should inform the employee that the result will be conveyed to the management and if the employee refuses to give his consent, the result regarding fitness may be reported to the authority. In all other circumstances, the doctor should not release information to the third party without the consent of the patient except when directed by a court of law.

The second problem arises when the doctor acting the traditional “doctor-patient role” has found that the employee is suffering from a condition, which may render him unfit to undertake certain work. The problem arises only when the employee refuses to give consent. The doctor should take sufficient time and trouble to explain to the patient in giving his consent and even if this fails, each case must be considered on its merits for disclosure.

## TRANSMISSION OF INFORMATION RESULTS OF STATUTORY MEDICAL AND BIOLOGICAL MONITORING

Notifiable diseases should be reported as per the law, individual employees should be informed of the results of their own tests and group results without individual identification can be given to the management to take preventive measures. Access to clinical records of an individual should not be granted as a right to other people whoever they may be or represent and whether or not they are professionally qualified.

## DISCOVERY OF NEWER HEALTH HAZARDS

The first and foremost duty of the physician is to inform the employer who in turn should inform the workman with the assistance of the doctor. Problems of ethics arise only if the employer is reluctant to permit such information to be divulged. If the evidence is clear and the management refuses permission for disclosure to enable workers to appreciate why precautions are necessary, doctors can resort to inform the workers himself; but before doing so, he should keep the management informed of the steps he proposes to take. The doctor should try his level best to persuade the management to reveal the information to workmen for the sake of prevention.

Like in other countries, associations like Indian Association of Occupational Health (IAOH) should formulate guidelines on Ethics, and it should be given to all its members & should be circulated to all the major industries to enable doctors to assimilate good ethical behavior.

Finally, the status of an occupational physician in an organization must be that of an impartial professional adviser, concerned mainly with safeguarding and improving the health of the employee. A professionally competent doctor with high levels of personal integrity would definitely be able to command the confidence of management & employees as well as resolve ethical conflicts amicably.

